# Assembly of early machinery for autophagy induction: novel insights from high resolution microscopy

**DOI:** 10.18632/oncotarget.13144

**Published:** 2016-11-06

**Authors:** Nicholas T. Ktistakis, Simon A. Walker, Eleftherios Karanasios

**Affiliations:** Signalling Programme, The Babraham Institute, Cambridge, UK

**Keywords:** autophagy, super resolution, autophagosome

The pathway of autophagy is conserved as a cellular response to starvation across eukaryotes [1]. Under conditions of nutrient limitation a novel double membrane organelle termed an autophagosome is formed that engulfs and delivers cytoplasmic material to the lysosomes for degradation and nutrient replenishment. Elaborations of this basic function include the autophagic elimination of aberrant intracellular membrane compartments and/or aggregated proteins *via* specific recognition systems [1].

Irrespective of whether the pathway targets non-specifically cytoplasmic proteins for degradation or it specifically recognises and eliminates defined cargo, a commitment step is the *de novo* formation of an autophagosome. Two mutually exclusive models of autophagosome formation can be envisaged: either the structure forms in isolation in the cytosol or in association with a pre-existing intracellular membrane compartment. The first model has given rise to the dominant view when autophagosome formation is depicted in diagrams (i.e. as an isolated structure forming *in vacuo*) but we and others have never seen it happening inside cells during live imaging [2, 3]. Instead, autophagosomes form always in association with some pre-existing membrane, be it the ER, mitochondria, Golgi, endosomes or the plasma membrane.

For a certain class of autophagosomes, association with the ER during their formation *via* an omegasome intermediate is a well characterised phenomenon [1]. In this view, elements of the ULK complex interact with the ER membrane in order to nucleate a pre-autophagosomal structure that very quickly matures into a PI3P-rich omegasome from where autophagosomes spawn. The exact membrane dynamics that underlie this simple description are likely to be extremely complex. Independent EM tomography studies have documented connection of the forming autophagosome with the ER *via* thin vesicular/tubular elements [4, 5], whereas similar careful analysis of the omegasome/ER morphology by CLEM has also described thin tubular extensions emanating from the ER and cradling the forming autophagosome [6].

What is even less clear than the omegasome-related dynamics is what happens at the earliest step which, in both yeast and mammalian cells, depends on the ULK complex and on ATG9 [1]. These early structures are not distinct enough to follow by conventional microscopy techniques and therefore ULK1/ATG9 “assemblies” cannot be distinguished from the background cellular “noise”. We addressed this problem by establishing that live cells during wide field imaging can be fixed and re-examined by high resolution optical techniques [7]. In this way, a newly formed ULK-containing autophagy structure whose provenance is known could be subsequently examined by SIM/dSTORM or by electron microscopy.

Using these techniques we found some interesting properties of the ULK1 complex as it nucleates early pre-autophagosomal intermediates [7]. The earliest structures, which based on our previous work are devoid of omegasome components [3], are small spherical particles approximately 20-30 nm in diameter and they sit on ER strands, frequently on extensions of the underlying ER. These ULK1 assemblies grow in size by addition of more small particles to a spherical structure of 300 to 400 nm in maximal diameter and resembling a virus particle in the arrangement of the spherical subunits (Figure 1). Association of these large spherical structures with the ER is evident even at the highest resolution afforded by dSTORM (Figure 1). In whole tomographic reconstitutions of these structures using FIB-SEM it is evident that they coincide with tubulovesicular regions in the vicinity of the ER resembling elements of the ERES or ERGIC. Importantly, despite the functional dependence of these structures on ER to Golgi traffic and their morphological resemblance to ERES or to ERGIC they are neither of these pre-existing organelles. Our working hypothesis is that these early structures are formed when elements of the early autophagic machinery generate autophagy-specific ERES-like sites which then grow into omegasomes by additional membrane re-arrangements and traffic. Given that a small number of ATG9-positive vesicles are always in the vicinity of these early structures and most of the time mark the ER sites from where they emerge [7], we also hypothesise that a sub-population of ATG9 may somehow mark the ER sites where these early ULK1- positive structures first coalesce. A speculative model is shown in Figure 1.

**Figure 1:**
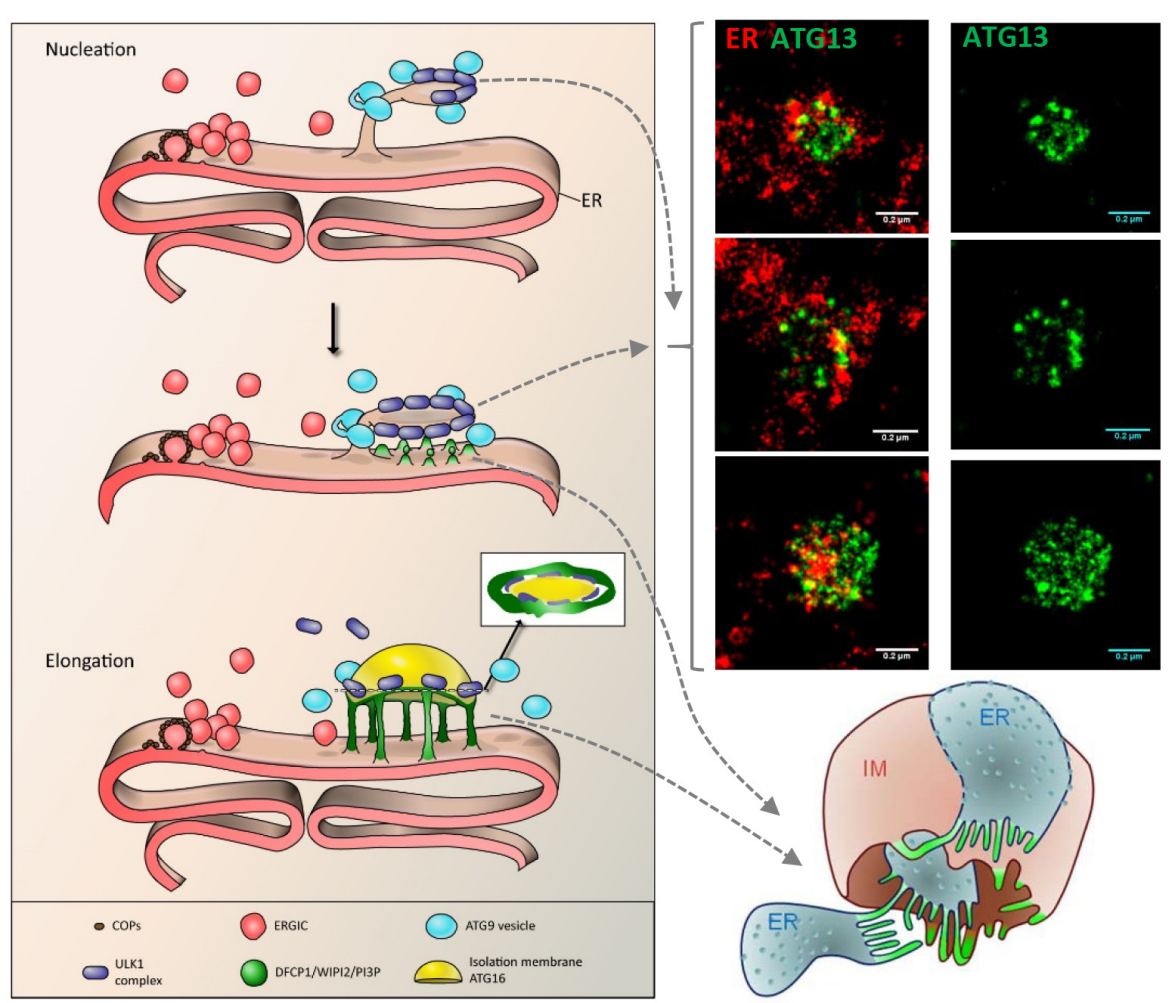
A speculative model of early autophagy steps Nucleation. A tubular extension of the ER together with a small number of ATG9 vesicles provide a platform for the assembly of ULK1 particles in an ordered arrangement. Such an arrangement is evident in dSTORM images of ER and ATG13, a component of the ULK1 complex (images to the right). The next step during nucleation involves formation of PI3P tubular extensions surrounding the ULK1 assembly. These extensions have been drawn to originate from the apposing ER to fit the model in Uemura et al. (model on bottom right reproduced with permission from [6]; omegasome-related tubules are depicted in green). Elongation. Continuous PI3P synthesis, LC3 lipidation and additional membrane supply (perhaps via ER derived vesicles amongst others) expands the site.

## ABBREVIATIONS

CLEM: correlative light electron microscopy
dSTORM: direct stochastic optical reconstruction microscopy
ER: endoplasmic reticulum
ERES: endoplasmic reticulum exit sites
ERGIC: endoplasmic reticulum-Golgi intermediate compartment
FIB-SEM: focussed ion beam scanning electron microscopy
SIM: structured illumination microscopy
PI3P: phosphatidylinositol 3-phopsphate
